# Healthy Diet and Lifestyle Improve the Gut Microbiota and Help Combat Fungal Infection

**DOI:** 10.3390/microorganisms11061556

**Published:** 2023-06-11

**Authors:** Samir Jawhara

**Affiliations:** 1 UMR 8576—UGSF—Unité de Glycobiologie Structurale et Fonctionnelle, Centre National de la Recherche Scientifique, F-59000 Lille, France; samir.jawhara@univ-lille.fr; Tel.: +33-(0)3-20-62-35-46; 2 Institut National de la Santé et de la Recherche Médicale U1285, University of Lille, F-59000 Lille, France; 3 Medicine Faculty, University of Lille, F-59000 Lille, France

**Keywords:** western diets, processed food, microbiota, dysbiosis, *Candida albicans*, smoking, excessive alcohol consumption, omega-3, vitamin D, vitamin E, micronutrients, probiotic, prebiotic, whole plant food

## Abstract

Western diets are rapidly spreading due to globalization, causing an increase in obesity and diseases of civilization. These Western diets are associated with changes in the gut microbiota related to intestinal inflammation. This review discusses the adverse effects of Western diets, which are high in fat and sugar and low in vegetable fiber, on the gut microbiota. This leads to gut dysbiosis and overgrowth of *Candida albicans*, which is a major cause of fungal infection worldwide. In addition to an unhealthy Western diet, other factors related to disease development and gut dysbiosis include smoking, excessive alcohol consumption, lack of physical activity, prolonged use of antibiotics, and chronic psychological stress. This review suggests that a diversified diet containing vegetable fiber, omega-3 polyunsaturated fatty acids, vitamins D and E, as well as micronutrients associated with probiotic or prebiotic supplements can improve the biodiversity of the microbiota, lead to short-chain fatty acid production, and reduce the abundance of fungal species in the gut. The review also discusses a variety of foods and plants that are effective against fungal overgrowth and gut dysbiosis in traditional medicine. Overall, healthy diets and lifestyle factors contribute to human well-being and increase the biodiversity of the gut microbiota, which positively modulates the brain and central nervous system.

## 1. Introduction

**Figure 1:**
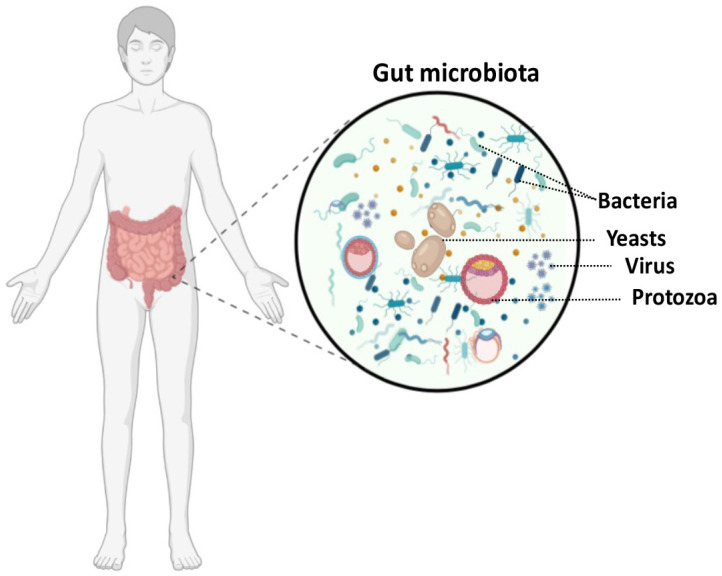
A schematic representation of the human gut microbiota, which contains 100 trillion microorganisms. These microorganisms, such as bacteria, viruses, fungi, and protozoa, are part of the gut microbiota.

The human gut microbiota consists of 100 trillion different types of microorganisms including bacteria, viruses, fungi, and protozoa [1,2]. Approximately 99% of gut bacteria belong to four phyla—Firmicutes, Bacteroidetes, Proteobacteria, and Actinobacteria [1,2]. A healthy individual’s gut microbiota is dominated by two phyla, Bacteroidetes and Firmicutes [3,4]. Different parts of the gastrointestinal tract contain different numbers and types of bacteria. Small numbers and few species live in the stomach and upper intestines, and bacteria gradually increase in number from the jejunum to the colon [5]. The gut microbiota is known to provide a variety of health benefits, including pathogen protection, nutrition, metabolism, and immune enhancement [6]. Human health depends on the symbiotic interactions between the human host and gut microbiota. However, dysbiosis occurs when the gut microbiota’s composition and function are adversely altered [3,4] (Figure 1).

The present review describes the role of different unhealthy dietary patterns and factors that promote *Candida albicans* overgrowth in the digestive tract and discusses how adherence to a healthy diet and lifestyle may help boost the immune response against *Candida* and reduce inflammatory diseases associated with gut dysbiosis. The review also focuses on the adverse effects of Western diets that are low in vegetable fiber and high in fat, which promote dysbiosis in the gut as well as fungal overgrowth, specifically *C. albicans*. In addition to an unbalanced diet, which is a risk factor for a large portion of the population and leads to the development of different diseases, other factors such as smoking, excessive alcohol consumption, lack of physical activity, prolonged use of antibiotics, and chronic psychological stress are associated with disease development and gut dysbiosis. This review also proposes strategies to improve lifestyles and unhealthy diets by reducing saturated/trans fats and increasing polyunsaturated fat, with special emphasis on omega-3 fatty acids. These strategies highlight the effects of whole plant foods with a moderate intake of dietary fiber, micronutrients, vitamins, fermented vegetables, probiotics, and traditional herbal medicines on gut dysbiosis and *C. albicans* overgrowth. These healthy diets and lifestyle factors have been shown to contribute to human health and well-being and help improve the gut microbiome, which in turn has a positive impact on the central nervous system and the brain (Figure 1).

## 2. Is Intestinal *Candida albicans* Colonization Linked to Inflammatory Diseases?

*C. albicans* is a commensal yeast that resides in the vagina and gastrointestinal tract of humans. *C. albicans* is an important cause of fungal infection worldwide [7]. This yeast can cause superficial, systemic, or invasive infections, with life-threatening outcomes [7]. Gut microbiota changes, epithelial barrier ruptures, and immune system dysfunction all facilitate the transition of *C. albicans* from the gut to vital organs [8]. Thus, the state of the gastrointestinal tract is a predisposing factor for life-threatening *C. albicans* infections that have high clinical and socioeconomic significance, representing one of the most commonly identified nosocomial pathogens. *C. albicans* interacts with its host through its cell wall, which is its main contact point [9,10]. The fungal cell wall protects yeast from environmental stress and maintains its cellular integrity. These stresses include drastic changes in temperature and osmotic pressure, dehydration, and the immune response [9,11]. Fungal cell wall components are critical for both morphogenesis and pathogenesis and could be targets for new antifungal drugs [9,11].

The cell wall of *C. albicans* is a dynamic structure composed of different layers [8,10]. This cell wall contains deep layers of chitin and intermediate β-1,3- and β-1,6-glucans in dense layers. Chitin is an N-acetylglucosamine homopolymer with a 1,4 linkage that folds anti-parallel and forms hydrogen bonds throughout its chains [12]. The inner skeleton of the *C. albicans* cell wall consists of chitin microfibril chains attached covalently to β 1,3-glucan [12]. Phosphopeptidomannan (PPM) covers the surface of the *C. albicans* cell wall but it is not covalently linked [13]. The *C. albicans* cell wall synthesizes β-mannosyls, which are associated with α-mannosyls in PPM. These mannosyls are present electively in a secreted glycolipid named phospholipomannan, which is associated non-covalently at the PPM cell wall surface [7,13]. Interaction of the *Candida* cell wall with the host cell is crucial for fungal colonization of the host and to activate different processes contributing to infection [7,13].

*C. albicans* overgrowth in the digestive tract is thought to be influenced by various factors, which are discussed below, especially prolonged antibiotic use and unhealthy dietary patterns. The morphological change from unicellular yeast to the filamentous hyphal form is crucial to the virulence of *C. albicans* [14]. In an infectious process, *C. albicans* yeast cells spread to other regions of their host via a phagocyte-dependent mechanism, in which neutrophils and macrophages facilitate dissemination while hyphae invade and damage host cells. The formation of hyphae within macrophages is associated with phagosome damage, which contributes to macrophage cell death by inhibiting phagosome acidification [14,15]. In terms of the role of T cell subsets in antifungal immunity, *C. albicans*-specific Th17 cells prevent fungal overgrowth and enhance epithelial barrier integrity in colonized tissue. In addition, IL-22 as well as IL-17A and IL-17F are produced by antifungal Th17 cells, which enhance the barrier function and protect the gut from intestinal injury [16]. Impairment of the IL-17/IL-22 pathway predisposes individuals to mucocutaneous candidiasis, but the same infection can also occur if type 17/type 1 immunity is impaired [17]. In relation to the role of IL-17 in social behavior, fungal gut colonization promotes murine social behavior both in the presence and absence of bacterial communities. These findings suggest that fungi are directly involved in the gut–brain axis via IL-17R-dependent signaling in neurons [18].

Another important aspect is that *C. albicans* colonization is involved in inflammatory diseases [19]. Experimental and clinical evidence suggests a possible link between *C. albicans* and Crohn’s disease (CD), which is a chronic transmural inflammatory bowel disease (IBD) [19,20]. CD usually affects the distal ileum and colon but may also affect other parts of the digestive tract [21,22,23]. High levels of antibodies to fungal cell wall glycans (known as anti-*Saccharomyces cerevisiae* antibodies or ASCA) capable of recognizing *C. albicans* are observed in CD patients. Furthermore, *C. albicans* is more frequently isolated from the stools of patients with CD. In an experimental setting, the role of *C. albicans* overgrowth in mucosal damage was assessed in a murine colitis model induced by dextran sulfate sodium (DSS) [20,24,25]. In this DSS model, *C. albicans* aggravated colonic inflammation in mice, and pre-inflammation of the colon induced by DSS promoted *C. albicans* overgrowth [20,26]. *C. albicans* overgrowth then led to the generation of ASCA, suggesting that circulating *C. albicans* mannan can trigger glycan antibody production in intestinal inflammation [20]. Like *C. albicans*, *C. glabrata* abundance was significantly elevated in mice with DSS-induced colitis [24,27]. This high abundance was associated with an increased inflammatory response and decreased microbiota biodiversity [28,29]. This indicates that *C. glabrata* abundance contributes to maintaining dysbiosis and inflammation in the gut [28].

## 3. Unhealthy Diets That Are High in Fat and Sugar with a Special Focus on Processed Food

Diets originating from some countries and culinary traditions, such as Mediterranean, Okinawa, and Nordic diets, all contain a large portion of whole plant foods (such as vegetables, fruits, and whole grains) and are associated with lower disease risks [30]. Western diets and lifestyles are spreading rapidly across the globe, resulting in a rapid increase in obesity and diseases of civilization (diabetes, cardiovascular diseases, cancer, and many more) [31]. In addition, unhealthy diets high in fat and sugar and especially processed foods consumed in Western countries induce changes to the gut microbiota associated with intestinal inflammation [32,33]. The processing of Western food includes hulling, heating, and the addition of preservatives, which all impact the microbes present in food [34]. The process of heating and the addition of preservatives may help to reduce pathogenic and spoilage bacteria, thereby ensuring safe food and extending the shelf-life. However, these measures of decreasing pathogenic or spoilage bacteria populations also decrease the intake of beneficial food-associated microbes [34]. Of note, processed food and high-fat diets are associated with dysbiosis, including a decrease in Bacteroidetes abundance and an increase in Firmicutes and Proteobacteria abundance in murine models [32,33]. This high-fat diet involves an increase in bacteria populations that produce lipopolysaccharides (LPS), such as Enterobacteriaceae, and a decrease in bacteria populations that suppress LPS, such as *Bifidobacterium* [32,33,35]. In addition, this diet affects the epithelial cells, causing alterations of the intestinal barrier including high intestinal permeability and increased LPS translocation from the gut to the bloodstream [36,37]. Increased consumption of fat, in particular long-chain fatty acids, can trigger the release of inflammatory cytokines by intestinal lymphocytes, intraepithelial lymphocytes, dendritic cells, and intestinal epithelial cells [36,38]. Diets low in fat but high in fiber have reduced inflammation and dysbiosis in patients with ulcerative colitis and improve their quality of life [39]. In their clinical study, Garcia-Gamboa et al. analyzed the cultivable fungal fraction in different samples from eutrophic, overweight, and obese individuals [40]. The number of fungal species in obese subjects was higher than in eutrophic subjects. Additionally, *C. albicans* was the most prevalent yeast present in obese subjects [40]. In terms of diets high in sugar, the consumption of simple sugars decreases the biodiversity of the gut microbiota and reduces luminal short-chain fatty acids (SCFAs) [41,42]. An experimental study showed that mice fed a high-sugar diet exhibited a significant increase in gut permeability, spleen weight, and neutrophil levels [43]. Following the administration of DSS, severe colitis rapidly developed in these mice. Furthermore, fecal samples from these mice showed a significant increase in pathobionts, such as *Escherichia coli* and *Candida* species [43]. 

Whole plant foods contain fermentable dietary fiber, which represents the fraction of food that is not digested by the endogenous enzymes present in the small intestine. In the large intestine, the microbiota can convert fermentable fiber into a variety of small organic metabolites, most notably SCFAs [44]. Acetate, propionate, and butyrate are SCFAs that exert anti-inflammatory, antioxidant, and anticancer properties [45,46]. For instance, among these whole food plants, whole-grain cereals have a complex dietary fiber composition such as arabinoxylans and β-glucan that contribute to enhanced SCFA production [47]. In a diet that is low in animal fat and protein but high in fiber, SCFAs provide colonocytes with additional energy. SCFAs bind to G-protein coupled receptors (GPCRs) such as GPR41 (free fatty acid receptor 3; FFAR3) and GPR43 (FFAR2), which are expressed on enteroendocrine L cells [48]. This in turn triggers the secretion of glucagon-like peptide 1 and peptide YY, which contribute to increased energy consumption, reduced food intake, and improved glucose metabolism and insulin secretion. A higher consumption of snack and junk food products is characterized by higher fecal levels of branched-chain fatty acids (BCFAs), which reflect bacterial catabolism of animal protein [49]. In this diet rich in fat and protein, bacterial enzymes first cleave complex proteins, releasing free amino acids and short peptides that undergo fermentation. An increase in protein fermentation, BCFAs (such as isobutyrate and isovalerate), organic acids, and gases cause dysbiosis and leakage of pathogen-derived compounds, including an increase in LPS levels in the blood, which lead to inflammation and insulin resistance [49]. With regard to the antifungal activity of SCFAs, the gut microbiota produces metabolites that have antifungal activity against *C. albicans* [50,51]. Both *C. albicans* growth and filamentation were reduced by sodium butyrate. In addition, the antimicrobial activities of macrophages were enhanced when exposed to *C. albicans* sensing [50]. Garcia et al. demonstrated that gut microbial metabolomes inhibit both *C. albicans* filamentation and fungal invasion of the human colonic epithelium [51].

## 4. Factors Contributing to Inflammatory Pathogenesis and Gut Dysbiosis

**Figure 2:**
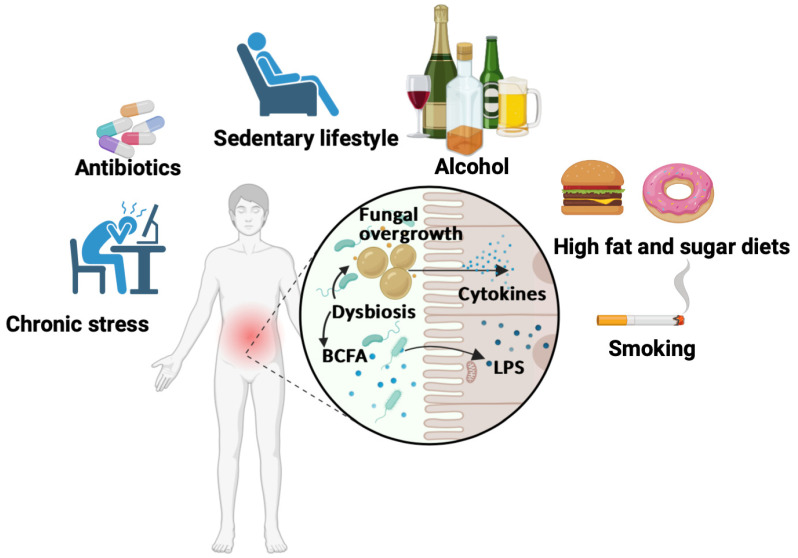
Schematic representation of unhealthy dietary patterns contributing to inflammatory pathogenesis and gut dysbiosis. Diets high in fat and sugar, smoking, excessive alcohol consumption, lack of physical activity, prolonged use of antibiotics, and chronic psychological stress all contribute to the development of gut dysbiosis. These diets cause an increase in branched-chain fatty acid (BCFA) production and populations of photobiont bacteria, such as *E. coli*, which produce lipopolysaccharides (LPS), as well as alteration of the intestinal barrier. Significant overgrowth of fungi is also observed, in particular *C. albicans,* in the gut during dysbiosis. All these factors induce the production of pro-inflammatory cytokines and leakage of pathogen-derived compounds, including LPS and fungal cell wall fractions, into the blood.

As discussed above, unhealthy dietary patterns are not the only lifestyle factors contributing to inflammatory pathogenesis; smoking, excessive alcohol consumption, lack of physical activity, prolonged use of antibiotics, and chronic psychological stress all contribute to disease development and intestinal dysbiosis (Figure 2). 

***Stress*** is a series of events that threaten the homeostasis of the body, resulting from either an external or internal factor (stressor). It has been shown that exposure to a social stressor modifies colon microbiota stability and leads to bacterial translocation and immunomodulation, with a decrease in the relative abundance of *Bacteroides* bacteria and an increase in the relative abundance of *Clostridia* bacteria [52]. An experimental study showed that rats infected with *C. albicans* and exposed to chronic varied stress exhibited an increase in fungal burden in the liver and kidneys [53]. In addition, macrophages produced significantly less nitric oxide, indicating that the chronic varied stress impaired the phagocytosis of *C. albicans* by macrophages [49,53].

***Excessive alcohol consumption*** is a major risk factor for many health problems. Chronic alcohol intake in mice increased mycobiota populations and facilitated the translocation of fungal β-glucan into the bloodstream, while treatment with antifungal agents reduced the intestinal fungal load, decreased β-glucan translocation, and ameliorated the liver damage caused by ethanol [54]. In a subsequent clinical study, patients with alcohol-associated liver disease had lower fungal diversity and showed an abundance of *Candida* species [55]. Furthermore, serum ASCA levels were associated with an increased mortality rate in patients with alcoholic hepatitis, indicating that treatment of intestinal fungi may benefit patients with alcoholic hepatitis and ASCA may help to predict their outcome [55].

***Prolonged use of antibiotics*** to combat infectious diseases is also a contributor to the modification of microbial richness and diversity (Figure 2). Some commensal and mutualistic bacteria are depleted after short-term treatment with broad-spectrum antibiotics in adults [56]. Antibiotics remove the protective microbiota, which makes the environment more conducive to *Candida* growth [57]. Spinillo et al. found that prolonged antibiotic use increased the risk of vulvovaginal candidiasis and that this risk was directly related to the duration of antibiotic use rather than the type of antibiotic [57]. In an experimental murine model, Bacteroidetes populations in mice were diminished long-term by antibiotic treatment, but the presence of *C. albicans* during antibiotic recovery promoted Bacteroidetes restoration [58]. However, *C. albicans* reduced *Lactobacillus* species over time and promoted the growth of *Enterococcus faecalis,* suggesting that an abundance of *C. albicans* maintains the perturbation of the bacterial microbiota in the gut [58]. A clinical study showed an increase in *C. albicans* levels in fecal samples from healthy volunteers receiving antibiotic treatment [59]. 

***Smoking*** reduces saliva production and changes the oral microbiota as well as increasing *Candida* colonization in the mouth [60]. Mun et al. showed that the risk of oral *Candida* infection was increased 7-fold in smokers [61]. The presence of oral *Candida* is also more likely in smokers with active carious lesions [62]. It is believed that smokers have a higher prevalence of *C. albicans* colonization due to a reduction in neutrophil activity against fungi [62]. In addition, smoking decreases the amount of gingival crevicular fluid, which contains antibodies and leukocytes. Mice deficient in IL-1β were highly colonized with *C. albicans* and showed lower survival rates when exposed to smoke than wild-type mice [63].

***Lack of physical activity*** contributes to several health outcomes and triggers the activation of different circulating markers of systemic inflammation (C-reactive protein, IL-6, TNFα, and neopterin) [64]. However, the regular practice of physical exercise improves quality of life, reduces systemic inflammation, and boosts the immune response against infection. Moderate exercise increased neutrophil phagocytic capacity against *C. albicans* in young men [65]. In line with this study, spontaneous mobility, chemotaxis, spontaneous attachment, and ingestion of *C. albicans* by macrophages significantly increased with physical training, supporting the advantageous effects of physical exercise on the phagocytic function of macrophages [66]. 

## 5. Strategies for Improving the Gut Microbiota and Reducing Fungal Load in the Gut

**Figure 3:**
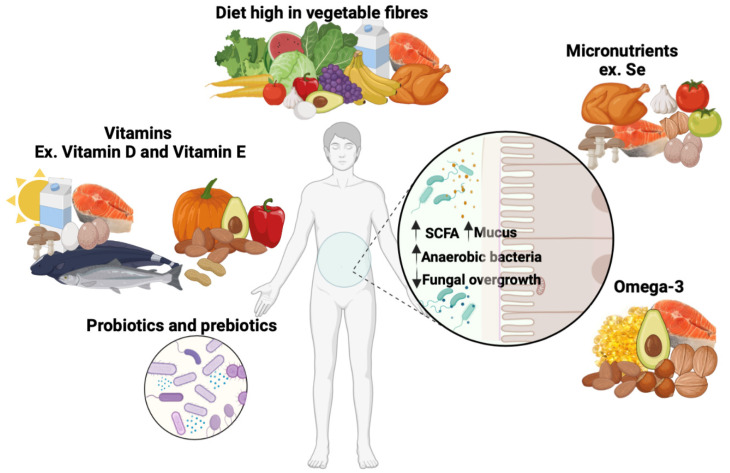
Schematic representation of the beneficial effects of a diet high in vegetable fiber, vitamins, micronutrients, omega-3 polyunsaturated fatty acids, and probiotics/prebiotics on the gut microbiota. A diet based on whole plant foods, including vegetables, fruits, whole grains, nuts, and seeds, associated with a portion of animal products (meat, dairy, and eggs) or seafood provides significant amounts of fiber, antioxidants, as well as vitamins like vitamins D and E, omega-3 fatty acids, and micronutrients such as selenium. In addition, a probiotic or prebiotic supplement is important to prevent gut dysbiosis. This diet contributes to an increase in short-chain fatty acid (SCFA) production and mucus secretion, as well as an increase in anaerobic bacteria such as *Lactobacillus* and *Bifidobacterium* species. Pathogenic fungi are significantly reduced in subjects consuming this diet. These conditions all promote gut microbiota biodiversity.

Low levels of vitamins D and E, trace elements such as selenium, and omega-3 polyunsaturated fatty acids are associated with negative clinical outcomes during infection [7]. A diversified diet rich in vegetable fiber, vitamins, and micronutrients associated with probiotic/prebiotic supplementation improves the gut microbiota and SCFA production and reduces fungal load (Figure 3). 

***Vitamin D*** is synthesized in the skin after exposure to the sun or is obtained from foods such as certain fish, egg yolks, dairy products, and mushrooms. In one clinical trial, a reduction in *Aspergillus fumigatus* induced IL-13 responses with daily vitamin D_3_ supplementation [67]. Vitamin D_3_ modulated cytokine responses towards an anti-inflammatory profile by inhibiting the expression of TLR2, TLR4, Dectin-1, and mannose receptors in peripheral blood mononuclear cells challenged with *C. albicans* [68]. Bouzid et al. showed that vitamin D_3_ acts as a fungicide and the liposoluble properties of this vitamin changed the integrity of fungal cell membranes, resulting in antifungal properties [69]. In line with this study, vitamin D_3_ impacted carbohydrate metabolism and ribosomal biogenesis in *C. albicans* [70]. In the murine intra-abdominal candidiasis model, vitamin D_3_ reduced the fungal burden in different organs. It also decreased the infiltration of inflammatory cells and levels of IFN-γ and TNF-α [70]. 

***Vitamin E*** is a fat-soluble compound found in many nuts, seeds, vegetables, and oils [71]. It has been shown that vitamin E has broad anti-inflammatory properties against *C. albicans* by suppressing NF-κB activity [72]. The addition of vitamins C and E to amphotericin B dramatically increased treatment efficiency in patients when compared to amphotericin B alone [73]. Furthermore, human red blood cells were protected against cytotoxicity caused by amphotericin B when vitamins C and E were added, showing that these vitamins, by their antioxidant properties, conferred protection against autoxidation induced by amphotericin B [73].

***Omega-3 polyunsaturated fatty acids*** contain α-linolenic acid (18:3 ω-3), stearidonic acid (18:4 ω-3), eicosapentaenoic acid (EPA; 20:5 ω-3), docosapentaenoic acid (22:5 ω-3), and docosahexaenoic acid (DHA; 22:6 ω-3) [74]. The blubber of marine mammals and liver from white fish contain long-chain fatty acids such as EPA and DHA [74]. Different studies have shown that EPA and DHA are beneficial not only to the health of the heart, brain, and eyes, but also to the immune system. There is evidence that polyunsaturated fatty acids and their ester derivatives are effective against a variety of oral pathogens, including *C. albicans* [75].

***Micronutrients*** such as selenium, which is a trace element, enter the food chain via plants consumed by humans and animals [76]. More than 25 selenoproteins incorporate selenium, which has antioxidant, chemopreventive, anti-inflammatory, and antimicrobial effects [77,78]. A selenium nanoparticle has been shown to adhere to *C. albicans* biofilms and then to penetrate this pathogen [79]. Thus, selenium nanoparticles damage *C. albicans* cell structure by substituting sulfur for selenium [79]. In selenium-deficient mice infected with *C. albicans*, their livers and spleens had significantly higher fungal loads than those of mice receiving selenium supplements [80]. In addition, selenium deficiency also impaired the ability of mouse neutrophils to kill *C. albicans* in in vitro experiments [80].

***Probiotic and Prebiotics***

Probiotics, including bacteria and yeasts, are defined as live microorganisms that have demonstrated beneficial health effects in humans (Figure 3). Current probiotics include lactic acid bacteria, *Bifidobacterium* species, and certain yeast species such as *Saccharomyces boulardii* or *S. cerevisiae*, all of which have been shown to be safe. Probiotics have been shown to reduce the symptoms of digestive disorders, such as irritable bowel syndrome, IBD, and infections with *Clostridium difficile*, as well as mood disorders such as depression [81,82,83]. Probiotics compete with pathogenic bacteria for nutrients and adhesion sites and ameliorate dysbiosis [81,84]. In addition, they improve epithelial lining barrier function, modulate the immune response, and indirectly influence other organs through neurotransmitter production and immune modulation [81,84]. It has been shown that *Lactobacillus* species decreased *C. albicans* biofilms during both the colonization and maturation phases of biofilm formation [85]. In addition, Lactobacilli may inhibit *C. albicans* growth through competition for nutrients and the production of lactic acid and other organic acids that reduce the pH [86]. Consequently, stress-related genes of *Candida* are upregulated in an unsuitable low-pH environment [86]. Furthermore, probiotics have been found to decrease the expression of genes related to ergosterol synthesis and fluconazole resistance efflux pumps [86]. In dysbiosis, probiotics could help restore healthy bacteria in the gut. 

Probiotics are vital to cross-feeding activity within microbial communities, influencing the metabolic capacity of other commensals [87]. Bifidobacteria can metabolize different types of carbohydrates from the host’s diet or mucosa, and their fermentation pathways produce different levels of acetic and lactic acids [88]. Bagarolli et al. showed that high-fat diets caused significant changes in the murine gut microbiota along with intestinal permeability, LPS translocation, inflammation, impaired glucose tolerance, and hyperphagia [89]. However, treatment with *Lactobacillus* species completely reversed these obesity-related features by changing the gut microbiota profile [89]. In line with this study, obese mice treated with *Lactobacillus* and *Bifidobacterium* strains had lower weights and cholesterol levels [90]. They also had fewer fungi, restored liver morphology, and beneficial modulation of the gut microbiota [90]. Brain activity can also be modulated by the consumption of selected probiotics [91]. Patients with chronic fatigue syndrome who received a *Lactobacillus casei* strain for 2 months showed significant increases in *Lactobacillus* and *Bifidobacterium* abundance as well as significantly decreased anxiety symptoms [91]. Anxiety and depression were alleviated in healthy volunteers taking *L. helveticus* R0052 and *B. longum* R0175 for 2 weeks [92].

Prebiotics, such as fructo-oligosaccharides, are non-digestible carbohydrates that are selectively metabolized by gut bacteria rather than being metabolized by the host [93] (Figure 3). Prebiotics are a dietary strategy that modifies the gut microbiota, both in composition and/or activity, to provide health benefits to the host [93]. In addition, prebiotics have been shown to attenuate the symptoms related to IBD and diarrhea caused by infectious and antibiotic agents [93,94]. Following DSS-induced colitis in mice, fructo-oligosaccharide supplementation diminished the pathological immune response and prevented structural impairment of the intestinal barrier [95]. Rousseau et al. showed that fructo-oligosaccharides were effective in preventing vaginal infections [96]. Different clinical and experimental studies explored the potential role of β-glucans (present in oats, barley, fungi, and some algae) and chitin (found in crustacean and beetle shells and the fungal cell wall) as prebiotics in the prevention of gut dysbiosis [97,98]. The colonization efficiency of *Bifidobacterium* strains was enhanced by feeding animals β-glucans and chitin in an in vivo model [99]. Oral administration of β-glucans or chitin to mice resulted in a decrease in aerobic bacterial populations, especially *E. coli* and *E. faecalis*, as well as fungal populations, while *Lactobacillus johnsonii* and *Bacteroides thetaiotaomicron* populations significantly increased in a DSS-induced colitis model [26,28,29]. It is clear from these studies that β-glucans or chitin play an important role in modulating the immune response and improving the biodiversity of the gut microbiota.

## 6. Plant and Food Compounds with Antifungal Properties

**Figure 4:**
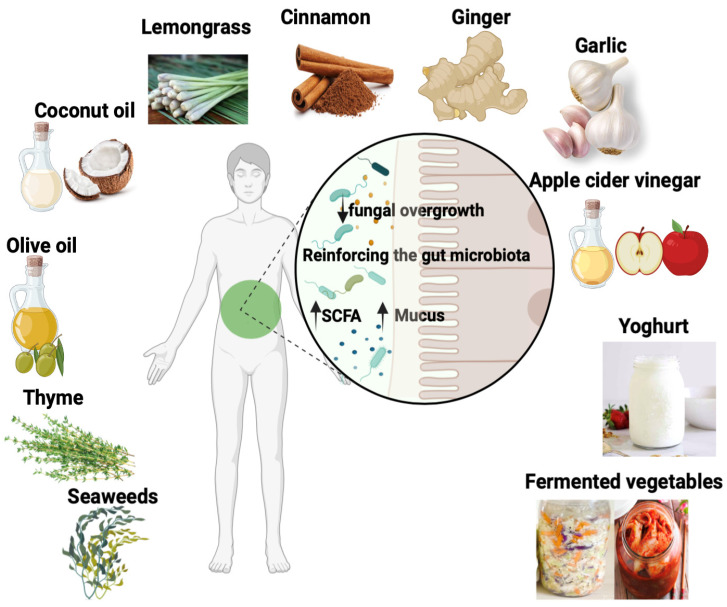
Schematic representation of the antifungal properties of natural compounds obtained from a variety of foods and plants known in traditional medicine. These foods and plants include garlic (*Allium sativum*), cinnamon (*Cinnamomum verum*), lemongrass (*Cymbopogon citratus*), coconut oil, ginger, seaweed (algae), thyme, olive oil, fermented vegetables, apple cider vinegar, and yogurt. These plants and foods reinforce the biodiversity of the gut microbiota, contributing to an increase in SCFA production and mucus secretion. In addition, pathogenic fungi are significantly reduced by these plants.

The antifungal properties of natural compounds obtained from a variety of foods and plants are known in traditional medicine. Numerous studies worldwide have analyzed the antimicrobial properties of these different plants, with many of them showing natural antifungal activity. These include garlic (*Allium sativum*), cinnamon (*Cinnamomum verum*), lemongrass (*Cymbopogon citratus*), coconut oil, ginger, seaweed (algae), thyme, olive oil, fermented vegetables, apple cider vinegar, and yogurt (Figure 4). 

***Garlic*** (*A. sativum*) belongs to the family Alliaceae and has been widely used for medicinal purposes for thousands of years. Freshly crushed garlic homogenates contain active components such as allicin, which has antibacterial and antifungal properties [100]. Allicin inhibits the growth of fungi by inhibiting succinate dehydrogenase [101]. The lipid composition of the outer surface of *C. albicans* has been reported to be affected by garlic [102]. In addition, garlic extract has been found to inhibit *C. albicans* growth by forming pits on the surface [101]. Low et al. showed that treatment with garlic extract prevented yeast cell transition to hyphae and SIR2 expression was downregulated when the garlic extract concentration was increased, indicating that garlic and its bioactive components suppressed *C. albicans* hyphae production and affected SIR2 gene expression [103]. The compound ajoene, derived from ethanolic garlic extract, inhibits the synthesis of phosphatidylcholine in cytosol and prevents the morphogenic transformation of fungi [104]. Recently, there have been several reports showing the therapeutic potential of *A. sativum* agglutinin (ASA), a lectin isolated from garlic bulbs [105,106]. Antifungal activity of ASA was found against different strains of *Candida glabrata* and *Candida auris*. Furthermore, fungal cells treated with ASA produced hydrogen peroxide and their cell integrity was affected by ASA treatment [105].

***Cinnamon*** (*C. verum*) is a spice found in the inner bark of cinnamon trees. It has anti-inflammatory, antimicrobial, antioxidant, and antiallergic properties. The main use of cinnamon in cooking is as a condiment and flavoring agent. Atai et al. evaluated *Absinthium artemisia*, eucalyptus, onion, cinnamon, turmeric, sage, mint, and *Calendula officinalis* against *C. albicans* strains and found that all eight exhibited antifungal properties. Compared to onion, mint, *C. officinalis*, and sage, cinnamon had higher potency and greater effectiveness, while turmeric, *A. artemisia*, and eucalyptus had similar effects [107]. To treat dental caries pathogens in India, dalchini (*Cinnamomum zeylanicum*; blume bark) is often soaked in water and used as a mouthwash [108]. *C. albicans* growth was prevented by dalchini extract for a longer period than with amphotericin B [108]. Additionally, cinnamon leaf and bark extracts inhibited *Fusarium graminearum*, *Fusarium proliferatum*, *A. fumigatus,* and *Trichophyton rubrum* [109]. Carvalho et al. found that cinnamon essential oils extracted from *C. zeylanicum* were effective in inhibiting several virulence factors of *C. albicans* clinical strains, including proteinase production, germ tube formation, and adhesion of *Candida* to buccal epithelial cells [110]. In vitro, cinnamon aldehyde and eugenol, which are the main constituents of cinnamon essential oils, prevented the growth of 80% of dermatophyte strains isolated from patients with dermatophytosis [111]. Tran et al. showed that both cinnamon bark and leaf essential oils were capable of exerting antifungal activity against *C. auris* and *C. albicans* by damaging the fungal membrane structure [112]. 

***Lemongrass*** (*Cymbopogon citratus*) is an aromatic plant widely distributed around the world. Lemongrass is a common food flavoring used in soups and teas. Lemongrass improves oral health, aids digestion, and controls bad breath [113]. Prajapati et al. showed that lemongrass oil and powder had antifungal activity against *C. albicans* [114]. Lemongrass essential oil at a concentration as low as 0.06% eliminated *C. albicans* [115]. Citral, the main component in this herbal essential oil (approximately 70%), exhibited efficient inhibitory effect against *Candida* suspensions [116]. *C. citratus* essential oil was investigated for its antifungal properties against *C. albicans* biofilms and was shown to reduce the viability of *Candida* cells in the biofilm [117]. As a vapor-phase agent, lemongrass essential oil showed antifungal activities against *C. albicans* by altering the fungal cell structure and surface morphology [118]. 

***Coconut oil*** contains monolaurin, a monoglyceride composed of lauric acid esterified with glycerol. Monolaurin has been shown to have broad bioactivities, such as antibacterial and antiviral properties [119]. Seleem et al. showed that oral *C. albicans* infection was significantly reduced by oral monolaurin treatment in mice [120]. In line with this study, treatment with monolaurin significantly reduced *C. albicans* biofilm formation in comparison with control groups [121]. In a murine experimental model, a coconut oil-rich diet reduced *C. albicans* colonization in mice compared to a diet containing beef tallow or soybean oil [122]. Adding coconut oil to the diet also altered the metabolic program of *C. albicans* cells [122]. Cecal contents of coconut oil-fed mice had fewer long-chain fatty acids than those of beef tallow-fed mice and the expression of several genes involved in fatty acid use was less active in *C. albicans* from coconut oil-fed mice than in *C. albicans* from beef tallow-fed mice [122].

***Ginger*** (*Zingiber officinale*) is an important spice consumed worldwide for culinary and medicinal purposes and it possesses multiple beneficial medical properties [123]. Regarding its antimicrobial properties, ginger extract exhibited anti-biofilm activity in bacteria [124]. 6-gingerols and 6-shogaol in ginger have been shown to inhibit biofilm and hypha formation in *C. albicans* [125]. Additionally, ginger extract inhibited the formation of biofilms by *C. albicans* and *Candida krusei* [126]. A synergistic effect was observed when ginger extract and fluconazole were administered together for the treatment of drug-resistant vulvovaginal candidiasis in mice, indicating that treatment of azole-resistant candidiasis was improved by co-administration of ginger extract and fluconazole [127]. 

***Seaweeds*** (algae) are predominantly aquatic photosynthetic organisms. Bioactive compounds found in seaweed have recently attracted the attention of researchers since they could contribute to the growth of the blue economy. Fathy et al. showed that mice infected with *C. albicans* and treated with seaweed extracts from Ulva fasciata Delile showed a reduction in histopathological change and a significant reduction in pro-inflammatory cytokine expression [128]. These data suggested that these seaweed extracts decreased the inflammatory response mediated by *C. albicans* through its active compounds, which enhanced cellular antioxidant defenses [128]. Seaweed *Gracilaria verrucosa* extract, which contains three steroids, terpenoids, and tannin, inhibited biofilm formation by *C. albicans* isolated from the saliva of a smoker [129]. Fucoidan, a form of sulfated polysaccharide found in marine algae, was found to have strong antifungal activity against *C. albicans* [130]. Phytotannins from the seaweed *Fucus spiralis* prevented the dimorphic transition of *C. albicans,* resulting in a reduction in *C. albicans* virulence and its ability to invade host cells [131].

***Thyme*** is used as both a culinary and medicinal herb. Both fresh and dried thyme leaves are consumed. There are several varieties of thyme used in culinary applications to enhance flavor. *Thymus vulgaris* is the most important species. Among the bioactive components of *T. vulgaris*, its essential oil contains thymol and carvacrol. Jafri et al. explored the synergistic interaction between thyme essential oils and antifungal drugs against *C. albicans* [132]. Thyme essential oils showed synergy with fluconazole against both planktonic and biofilm-forming *C. albicans* and *Candida tropicalis* [132].

***Olive oil*** plays an important role in the Mediterranean diet and enhances important stimuli for bowel movements through interactions with bile acids [133]. Olive oil mainly contains oleic acid (18:1), which accounts for 55–83% of its fatty acid composition. It also contains linoleic and linolenic acids. Administration of extra-virgin olive oil improved intestinal permeability and alleviated inflammation-related histopathological features [134]. An experimental study showed that oleic acid exhibited antifungal activity against *C. albicans* [135]. 

***Fermented vegetables*** contain a wide variety of bacteria that are involved in digestion, antipathogenic, and immunomodulatory activities [136,137]. The natural bacteria found on vegetable surfaces serve as starter cultures for fermentation. Vegetable carbohydrates are then used and converted into lactic acid by these bacteria [138]. The fermentation process produces other compounds, such as carbon dioxide, acetic acid, and bioactive substances [138]. During this fermentation process, microbial cells multiply rapidly and the ecosystem changes over time, with different species dominating at different stages of fermentation [139,140]. *Lactobacillus plantarum*, *Lactobacillus brevis*, and *Lactobacillus sakei* are among the lactic acid bacteria found in sauerkraut and kimchi ferments [139,140]. These bacteria provide immunomodulatory signals, support digestive processes, produce bioactive compounds (e.g., isothiocyanates), and suppress pathogens by producing acids and bacteriocins. Several bacterial isolates from sauerkraut or kimchi have been shown to act as antimicrobials against pathogenic fungi [141,142]. Three *L. plantarum* strains isolated from kimchi showed antifungal activity against *C. albicans*, as evidenced by a significant decrease in fungal growth [141]. Furthermore, *Lactobacillus* products from kimchi boosted mucosal immunity by enhancing secretory IgA levels in mice and displayed anti-*C. albicans* activity [142]. 

***Apple cider vinegar*** (ACV) is made from cider that has been transformed by a process known as acetous bioconversion. It has low acidity (5% acetic acid). Flavonoids and polyphenols are also present, as well as vitamins, minerals, and organic acids. In terms of the role of ACV in boosting the immune response, Yagnik et al. showed that ACV increased the phagocytic activity of monocytes against *C. albicans* [143]. ACV is also able to reduce *C. albicans* viability and growth [143].

***Yogurt*** is fermented milk acidified with viable and well-defined bacteria (*Lactobacillus bulgaricus* and *Streptococcus thermophiles*) [144]. In addition to supplying highly bioavailable protein, yogurt also provides an excellent source of calcium as well as probiotic bacteria, making yogurt a good component of a healthy lifestyle [144]. The daily consumption of yogurt, enriched with live *L. acidophilus*, led to the disappearance of recurrent *Candida* vaginitis in comparison to pasteurized yogurt [145]. Additionally, a correlation was found between anti-*Candida* activity and the presence of acetic acid bacteria in dairy associations [146]. Hu et al. showed that the consumption of probiotic yogurt reduced fungal colonization in women [147].

## 7. Conclusions

The busy routines of everyday life have prompted many populations to adopt Western food diets that are high in fat and sugar and have led to many individuals to adopting sedentary routines within an environment that lacks sunlight. In addition, our daily routines often cause us to experience chronic psychological stress, which in turn leads to the development of unhealthy habits such as smoking and drinking alcohol, as well as a lack of socialization. The factors listed above have a serious impact on our intestinal microbiota, promoting gut dysbiosis that includes an increase in opportunistic *C. albicans* overgrowth and a decrease in anaerobic bacteria populations such as *Bifidobacterium*. However, there is now increasing evidence that healthy food choices and a healthy diet rich in fiber can help prevent many chronic inflammation-related diseases such as IBD. Various approaches have been discussed in this review that highlight the factors responsible for intestinal dysbiosis and the overgrowth of pathogenic yeast in the gut. The review also suggests how healthy dietary patterns may be able to improve the biodiversity of the gut microbiota and presents an overview of a variety of foods and plants that are known to be effective in traditional medicine for fighting fungal overgrowth and gut dysbiosis. Taking all of these factors into account, evidence suggests that healthy diets and lifestyle factors are responsible for improving human well-being as well as increasing the biodiversity of the gut microbiota, which in turn has a positive effect on the central nervous system (CNS) and brain. Of note, CNS disorders are often associated with inflammatory processes, whereas a diet that consists of a low intake of ultra-processed foods and a high intake of plant foods is gaining increasing attention as a potential therapeutic strategy. The emergence of the new research field “nutritional psychiatry” offers promise in identifying dietary components that are truly important for mental health (including mood disorders such as anxiety, depression, and other neuropsychiatric conditions), their impact on preventing or treating these disorders, as well as how they influence the gut microbiota.

## Data Availability

Not applicable.
